# A network-based approach to disturbance transmission through microbial interactions

**DOI:** 10.3389/fmicb.2015.01182

**Published:** 2015-10-27

**Authors:** Dana E. Hunt, Christopher S. Ward

**Affiliations:** ^1^Marine Laboratory, Duke University, Beaufort, NC, USA; ^2^Integrated Toxicology and Environmental Health Program, Duke University, Durham, NC, USA

**Keywords:** interaction networks, disturbance, phytoplankton, anthropogenic, storms

## Abstract

Microbes numerically dominate aquatic ecosystems and play key roles in the biogeochemistry and the health of these environments. Due to their short generations times and high diversity, microbial communities are among the first responders to environmental changes, including natural and anthropogenic disturbances such as storms, pollutant releases, and upwelling. These disturbances affect members of the microbial communities both directly and indirectly through interactions with impacted community members. Thus, interactions can influence disturbance propagation through the microbial community by either expanding the range of organisms affected or buffering the influence of disturbance. For example, interactions may expand the number of disturbance-affected taxa by favoring a competitor or buffer the impacts of disturbance when a potentially disturbance-responsive clade’s growth is limited by an essential microbial partner. Here, we discuss the potential to use inferred ecological association networks to examine how disturbances propagate through microbial communities focusing on a case study of a coastal community’s response to a storm. This approach will offer greater insight into how disturbances can produce community-wide impacts on aquatic environments following transient changes in environmental parameters.

## Microbes as Important Responders to Ecosystem Changes

Most people and development reside near water bodies, so human activities profoundly affect both freshwater and marine ecosystems (Vitousek et al., 1997). In these aquatic environments, microbes are the numerically- and often the biomass-dominant organisms, thus how they respond to anthropogenic impacts determines both ecosystem health and biogeochemical rates. Although a large body of research explores microbial responses to long-term human alteration of the environment (e.g., climate change, ocean acidification), here we focus on pulse disturbance events that disrupt “ecosystem, community, or population structure and [change] resources, substrate availability or the physical environment” (White and Pickett, 1985). High levels of diversity and short generation times make aquatic microbes a sensitive model system to explore disturbance, but also complicate tracking the impacts and progression of disturbance. The wide range of pulse disturbances affecting aquatic environments including storms, snowmelt, mixing/upwelling, and chemical or sewage spills allows microbial ecologists to probe community responses to environmental changes.

In general, microbial communities are not resistant, which is defined by Allison and Martiny (2008) as the degree to which microbial composition remains unchanged in the face of disturbance. This low resistance is likely due to the wide range of genetic and physiological targets present in diverse microbial communities as well as microbes’ short generation times, which allow observation of both positive (increased growth) and negative (death, impaired growth) responses. Following a disturbance, community resistance and resilience are generally determined by comparing community composition at specific time points (Shade et al., 2012). A metric of community recovery, microbial resilience is generally defined as a return to the initial community composition (Allison and Martiny, 2008; Shade et al., 2012). However, aquatic microbial communities are highly dynamic and continually change in response to seasonal environmental variables (e.g., light and temperature) or subsequent disturbances (Chow et al., 2013; Needham et al., 2013; Yung et al., 2015). Thus, we define the resilience of an aquatic microbial community as the rate at which the community composition returns to a *non-disturbed* state following a disturbance. This definition of resilience requires understanding the disturbance-independent temporal dynamics of microbial communities. Although marine microbial communities exhibit regular seasonal patterns at monthly timescales (Fuhrman et al., 2006; Gilbert et al., 2012; Giovannoni and Vergin, 2012), high resolution and repeated annual sampling reveals shorter-term and inter-annual variability at the days to weeks time scale of disturbance responses (El-Swais et al., 2015), complicating differentiation of disturbance responses from annual patterns and stochasticity. However, even if the seasonal community trajectory is known, challenges to measuring microbial responses to disturbance include confounding factors such as unrelated changes in environmental variables, stochasticity in response and recovery, dispersal limitation, genomic evolution to become resistant to disturbances, and microbial interactions with other organisms (Shade et al., 2012; Nemergut et al., 2013). We propose to begin addressing the importance of microbial interactions to gain new insights into the mechanisms underlying the resistance and resilience of microbial communities.

## Microbial Interactions in Community Assembly

As identifying the drivers of microbial community composition is complex, most investigators first consider environmental selection, and generally secondarily address other aspects of community assembly: dispersal, drift (stochasticity), and diversification (mutation; Vellend, 2010; Hanson et al., 2012; Nemergut et al., 2013). However, dispersal may limit the viable population present even when conditions favor growth (Caporaso et al., 2011; Hanson et al., 2012) or alternately, environmental changes may not persist long enough for viable cells to respond (Hutchinson, 1961). An emphasis on deterministic processes also ignores the role of stochasticity in community assembly and the potential for communities with different compositions to carry out the same processes at the same rates (e.g., functional redundancy; Werner et al., 2011; Bissett et al., 2013; Hellweger et al., 2014; Zhou et al., 2014). Further, microbial genomes evolve in response to disturbance; they can develop resistance to disturbances such as antibiotics or heavy metals, alter metabolic capabilities, and change physiological niche width (Riehle et al., 2003; Davies and Davies, 2010). Although microbial communities are shaped by a combination of selection, drift, dispersal and evolution, there is value in addressing subsets of these factors, here we focus on selection via biological interactions following disturbance.

Microbial ecology research currently emphasizes the role of interactions in the community response to environmental changes and disturbances (Faust et al., 2012; Bissett et al., 2013; Fuhrman et al., 2015). Although some examples of relationships between specific taxa and environmental variables exist (Field et al., 1997; Johnson et al., 2006; Yung et al., 2015), interactions between aquatic microbes have not been well explored. Even for predation by viruses and grazers, one of the best studied microbial interactions, much still remains to be discovered about the interaction specificity (Sullivan et al., 2003; Apple et al., 2011). Furthermore, the nature of biological interactions may be dictated by characteristics of dominant aquatic bacteria; the most abundant marine populations (e.g., *Pelagibacter*, *Prochlorococcus*) are known for their streamlined genomes, small cell sizes, and efficient use of resources (Giovannoni et al., 2014). Some of the evolutionary success of these organisms may be due to their conservation of limited resources by shedding genes encoding critical functions and outsourcing these functions to other members of the community (Black Queen Hypothesis; Morris et al., 2012). For example, both *Pelagibacter* and *Prochlorococcus* have lost the gene for catalase which protects cells from hydrogen peroxide; as hydrogen peroxide diffuses through cell membranes, other members of the microbial community can protect catalase non-producers (Morris et al., 2011, 2012). Yet aquatic organisms with complex genomes have also evolved required interactions with other organisms; many eukaryotic algae have a B_12_-dependent methionine synthase rather than the B_12_-independent version, despite the fact that B_12_ is only synthesized by prokaryotes. This suggests that interactions with other organisms evolve due to net ecological advantage rather than solely genome streamlining.

Although outsourcing key requirements may be ecologically advantageous, long distances between cells, on average ∼100 μm (Hunt et al., 2010), may exclude specific types of biological interactions for planktonic organisms such as syntrophy where physical coupling allows efficient transfer between cells (Boetius et al., 2000; Malfatti and Azam, 2009). For truly free-living organisms, interactions likely involve diffusible compounds, suggesting that interaction partners may not be highly specific or involve complex regulation. Experimental evidence supports complementation of lost capabilities by non-specific interaction partners: a range of reduced sulfur sources can be used by SAR11 (Tripp et al., 2008) and many bacteria can provide B_12_ for auxotrophs (Croft et al., 2005). Additionally, some obligate relationships, at least in artificial laboratory conditions, do not involve regulation or signaling (Durham et al., 2015), while others are regulated (Kazamia et al., 2012), suggesting a number of potential strategies for interactions. Although outsourcing key functions is thought to be evolutionarily adaptive, interactions also incur costs: B_12_ additions have been shown to stimulate phytoplankton, implying that an interaction limits algal growth (Sañudo-Wilhelmy et al., 2006; Bertrand et al., 2007). While experimentally-verified interactions between microbes remain rare, the success of aquatic organisms may stem at least partially from outsourcing key functions. Thus, increasingly, microbial ecologists are incorporating interactions into our understanding of microbial communities, including interaction-mediated transmission of disturbance, resistance, and resilience.

## Using Association Networks to Explore Disturbance

In general, microbial interactions cannot be directly observed, thus ecological relationships are instead inferred based on environmental observations of co-occurrence patterns and synchronous population dynamics (Ruan et al., 2006; Steele et al., 2011; Faust et al., 2012). Patterns of microbial relative abundance obtained from communities sampled over spatial or temporal gradients are used to generate correlation-based association networks of potential interactions between operational taxonomic units (OTUs) and between OTUs and environmental variables (Barberan et al., 2012; Faust et al., 2012; Fuhrman et al., 2015). These correlations are interpreted to capture biological mutualisms such as cross-feeding and exchange of metabolites (Kazamia et al., 2012; Morris et al., 2012), functional redundancy (Eiler et al., 2012; Needham et al., 2013), or antagonism through competition or predation (Pernthaler and Amann, 2005). In addition to the well-known biases of DNA extraction, PCR amplification and in inferring patterns of organismal abundance from library relative abundance data (Polz and Cavanaugh, 1998; Acinas et al., 2004; Friedman and Alm, 2012), association networks also suffer from a number of network-specific limitations. First, association networks assume that 16S rRNA-based OTUs are ecologically coherent in spite of known microdiversity (Hunt et al., 2008) and physiologically identical under all environmental conditions, e.g., does not account for phenotypic plasticity based on environmental conditions (Nemergut et al., 2013; Worden et al., 2015). Second, associations may serve as proxies for specific environmental conditions or niches rather than indicating true interactions (Fuhrman et al., 2015). Finally, metrics of association strength are not standard and depend on the metric chosen, number of samples, taxa relative abundance, beta diversity, and data normalization (Ruan et al., 2006; Faust et al., 2012; Friedman and Alm, 2012; Berry and Widder, 2014). Currently, this field also lacks methods to add additional support for interactions such as observed physical associations to networks (Malfatti and Azam, 2009; de Vargas et al., 2015). While acknowledging the limitations of correlation-based association networks, we believe this technique has the potential to inform our understanding of aquatic microbial community dynamics.

Recently, association networks were employed to predict the bacterial response to disturbance (Bissett et al., 2013); expanding on this work, we propose to use network approaches to quantitatively examine the importance of interactions in altering the taxa affected by disturbance. Of particular promise are techniques developed in information technology and social learning, where interactions transmit signals between nodes, much in the same way that initial disturbance-induced changes in an OTU’s abundance may in turn affect the abundance of its interaction partners at later time points. One technique to look at disturbance transmission, information flow analysis can model the transmission of disturbance through the interaction network using the interaction strength and considering all possible paths in a network (Missiuro et al., 2009). Information flow analysis accounts for the strength of inferred interactions, enabling prediction of how changes in the relative abundance of a specific organism or value of an environmental variable will affect the microbial community, and thus provides a metric of predicted community resistance. Additionally, network-based diffusion analysis could be used to determine quantitatively whether association networks help to explain the propagation of disturbance through the community (Franz and Nunn, 2009). Operationally, association networks would be used to predict the temporal dynamics of microbial community composition following disturbance. The effects of disturbance on the rest of the community (changes in OTU relative abundances) can be predicted using information flow analysis. This predicted community composition would be compared to the actual community composition following a disturbance and community changes predicted from a randomized network generated by preserving the association network topology but repeatedly, randomly assigning OTUs to network nodes. Thus if the association network’s inferred interactions are truly important in the community’s disturbance response, the true association network should more closely match the observed community responses compared to a set of randomized networks. These methods will quantify the importance of interactions and predict community responses to specific environmental conditions, enhancing our understanding of the role interactions play in disturbance.

Although these techniques are potentially powerful methods to track community responses to disturbance, there are a number of logistical considerations in using association networks to follow the propagation of disturbance through microbial communities. First, network-based analyses require large datasets both pre- and post-disturbance synoptic with community changes to develop an association network and track the disturbance response, respectively. As disturbance-responsive taxa are often rare, they may not be well-represented in association networks which generally require taxa to be present in most samples (Shade et al., 2014). Moreover, taxa which can respond quickly to environmental changes may exhibit fewer, or different types of biological interactions than the streamlined genome oligotrophs which dominate many aquatic environments (Polz et al., 2006). Additionally, microbial community composition, generally measured using small subunit ribosomal RNA genes, may not be sufficiently sensitive to detect a disturbance response due to the time for cells to reproduce or predation of responsive taxa, necessitating the use of alternative metrics such as activity measurements (Berga et al., 2012; Hunt et al., 2013). Finally, dispersal may limit the response of taxa even under conditions which favor growth. With the relatively short time scales of pulse disturbances, it may be necessary to include prior relative abundance in predicting an OTU’s potential responsiveness to disturbance. With all of these caveats in place—we suggest first studying time periods when disturbances are predicted to produce large changes in the microbial community.

Theoretically, anthropogenic disturbances should have the greatest impact when highly-connected taxa change their abundance or activity. Research on networks has shown that disturbances that target central “keystone” nodes dramatically alter the rest of the network (Albert et al., 2000; Montoya and Solé, 2002). Ecology posits the existence of keystone taxa—which may impact multiple members of the community through either positive interactions (production of substrates or co-factors utilized by other microbes) or competitive exclusion, predation, disease, or habitat modification (Power et al., 1996). Keystone organisms are often defined as those with disproportionate ecological roles given their relative abundance (Power et al., 1996); however as microbial ecology lacks techniques to remove specific OTUs and quantify the ecosystem effect, here we operationally define keystones as taxa located at the hubs of association networks with an increased number of network connections relative to abundance (high mean degree); however, other metrics take into account the betweenness and closeness centralities of the node as well as strength of interactions (Missiuro et al., 2009; Bissett et al., 2013; Berry and Widder, 2014; Peura et al., 2015). Yet many network hubs may be artifacts of network construction rather than true keystone taxa (Berry and Widder, 2014). Although the concept of keystone taxa has not been thoroughly explored in microbial ecology, previous studies have suggested that microbial community activity and succession is driven by interactions with phytoplankton (Azam et al., 1983; Kent et al., 2007). The factors that promote phytoplankton growth are generally well known: light, inorganic nutrients, specific temperature ranges; and phytoplankton are the dominant primary producers in most aquatic systems. These photosynthetic organisms shape the microbial community through primary production, but at the same time outsource the production of essential functions (e.g., hydrogen peroxide detoxification) to the broader community (Cole, 1982; Kazamia et al., 2012; Morris et al., 2012). Other taxa interact with phytoplankton through photosynthate consumption, degradation of detrital material, symbiosis, and predation (Croft et al., 2005; Stocker et al., 2008; Morris et al., 2011; Teeling et al., 2012; Durham et al., 2015). Finally, phytoplankton serve as hubs in association networks (Steele et al., 2011) and could function as keystone organisms in aquatic ecosystems. While the ecological role of some potential keystone taxa has been identified, e.g., nitrogen-fixing bacteria (Tyson et al., 2005), for most network hubs there is no known keystone function (Steele et al., 2011; Bissett et al., 2013). Thus the phytoplankton, where growth-promoting factors and relationships with other microbes are relatively well-characterized, represent an ideal model system in which to explore the biological interactions that underlie association networks during pulse disturbances.

## Using Storms to Explore Disturbance Propagation

Storms represent complex, pulse disturbances that integrate both natural and human impacts. Storm-driven rain and wind events increase turbidity and introduce nutrients, organic material, and microbes from both the benthos and land into aquatic systems; while anthropogenic activity increases nutrient fluxes, impacts the timing of freshwater inputs, and contributes other chemical pollutants. Thus storms are multi-faceted disturbances; yet, unlike some discrete disturbances (e.g., Deepwater Horizon oil spill), they occur frequently enough to allow comparison across different storms, environments, and microbial communities (Berga et al., 2012; Yeo et al., 2013). Here we use storms as a model disturbance to explore using association networks to track the propagation of disturbance through the microbial community.

**FIGURE 1:**
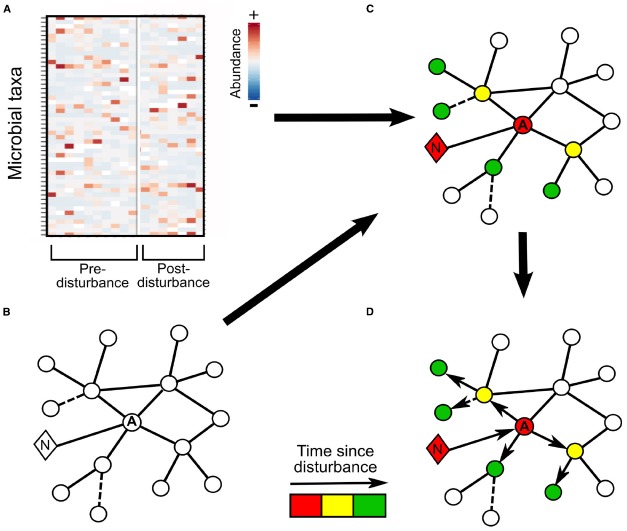
**Schematic for tracking disturbance transmission through a microbial community. (A)** Repeated community observations pre-disturbance are used to develop **(B)** a correlation-based association network for the microbial community. The circles represent operational taxonomic units (OTUs), with the keystone algal OTU denoted with an A, the diamond represents nutrients and is labeled with an N, solid lines connecting shapes indicate statistically significant positive correlations and dashed lines negative correlations between the connected taxa or environmental parameters. The same environment is intensively sampled following a storm to track short-term alterations in environmental variables and community composition. **(C)** The post-disturbance community composition from three time periods is overlaid onto the interaction network to track the propagation of disturbance through the community: the red coloring indicates the changes directly following the storm: an increase in nutrients and shortly thereafter increased algal abundance. Yellow coloring indicates OTUs which display relative abundance changes at the second time point following disturbance and green those OTUs which change in relative abundance in the final period. **(D)** Arrows indicate the direction of inferred disturbance propagation through the network based on the timing of observed changes in OTU relative abundance.

To investigate this concept further, we will follow the progression of storm-mediated impacts on a simplified microbial community association network where an alga serves as a keystone microbe and a network hub. In our model system (Figure 1), the major storm impact is an increase in nutrients (Iluz et al., 2009; Johnson et al., 2013); and the first microbial community responder is the keystone algal OTU, which is positively correlated with nutrient levels. Using association networks prepared from non-disturbance data (Figures 1A,B), we can infer which other OTUs are likely to respond to a change in algal abundance. With high resolution post-storm sampling, we can observe changes in OTUs correlated with the early responders, as shown by lines (edges) connecting these taxa to the alga, which should exhibit changes in activity or relative abundance at intermediate time points if that OTU is dependent on the alga, e.g., through metabolism of photosynthate (Figures 1C,D: yellow circles). At still later time points, the disturbance may propagate to taxa which interact with the yellow OTUs (Figure 1D: green circles). Alternately, at this same time point, OTUs with inferred relationships with the alga, but utilizing detritus associated with bloom termination rather than photosynthate from active algal cells may exhibit increases in relative abundance (Figures 1C,D: green circle; Teeling et al., 2012). Thus network-based approaches can offer biological insights into phytoplankton-bacterial interactions, the propagation and persistence of disturbance (Figure 1), and community stability (Carpenter et al., 2011; Veraart et al., 2012). Even anecdotal observations of how OTUs respond to disturbance can generate hypotheses that can be verified using more controlled laboratory or manipulation experiments.

Here, we have presented a cartoon storm as a pulse of nutrients, in reality storms and other ecological disturbances are complex. In addition to nutrients, storms introduce human pollutants into aquatic ecosystems, including pesticides, oil, untreated human waste, etc., that will have direct and interaction-mediated effects on the microbial community. Unlike our simple example in Figure 1, there may be multiple, competing impacts on our keystone algal OTU. For example, chemical herbicides such as atrazine impact phytoplankton due to the conservation of photosystem II between cyanobacteria, algae, and plants (Huber, 1993). While, the specific impacts of most chemicals are correlated with concentration; another herbicide class of synthetic auxins (e.g., 2,4-dichlorophenoxyacetic acid) is toxic to cyanobacteria at high concentrations but stimulates growth at lower levels (Mishra and Pandey, 1989), a subtlety which is not readily incorporated into association networks. Among other anthropogenic pollutants, fungicides are generally less specific than herbicides, targeting highly-conserved cellular processes such as respiration and thus directly affect a range of microbes (Casida, 2009; Yang et al., 2011). Thus, along with nutrients, storms introduce a cocktail of chemicals to aquatic environments, complicating evaluation of direct and indirect community effects on the microbial community.

## Conclusions

Here, we discuss the potential for association networks to track the propagation and persistence of disturbance in a microbial community. We have identified two major opportunities afforded by this approach: (1) to quantify the importance of interactions in a microbial community’s response to disturbance and (2) to generate biological hypotheses about the network’s inferred interactions. However, a major challenge of this approach is that to characterize a microbial community’s resistance and resilience we first need to understand disturbance-independent microbial community dynamics (Shade et al., 2012), suggesting the need for long-term monitoring of key study sites. Although the vast amounts of data required can appear daunting, specific taxa have been shown to repeatedly respond to storms (Jones et al., 2008) and the field is beginning to identify general characteristics of disturbance-responsive organisms (Shade et al., 2014), suggesting that there are conserved rules that govern microbial communities’ disturbance responses. However, to tease apart the effects of factors that tend to co-vary in the environment, for example, separating the stimulatory effects of increasing nitrogen versus organic carbon, there is an additional role for controlled, replicated manipulations of natural aquatic communities. Beyond community changes, these experiments will also provide predictions about the alteration and restoration of ecosystem function following a disturbance, either by linking specific taxa to functions or by identifying the types of disturbance which may be most likely to disrupt specific processes (Amend et al., 2015). An association network-based approach to analyzing microbial community disturbances and experimental manipulations will provide a basis to mechanistically predict community response to both pulse and press environmental changes.

### Conflict of Interest Statement

The authors declare that the research was conducted in the absence of any commercial or financial relationships that could be construed as a potential conflict of interest.
